# Blood glutamine synthetase signaling in alcohol use disorder and racial disparity

**DOI:** 10.1038/s41398-022-01837-w

**Published:** 2022-02-22

**Authors:** Lailun Nahar, Sarah E. Kaufman, Patrick G. Davis, Stephanie L. Saunders, Elizabeth A. Disbrow, James C. Patterson, Hyung W. Nam

**Affiliations:** 1 Department of Pharmacology, Toxicology, and Neuroscience, Shreveport, LA 71130 USA; 2 Department of Psychiatry and Behavioral Medicine, Shreveport, LA 71130 USA; 3 Department of Neurology, Louisiana State University Health Sciences Center, Shreveport, LA 71130 USA

**Keywords:** Prognostic markers, Predictive markers

## Abstract

As of 2018, 14.4 million adults ages 18 and older in the U.S had alcohol use disorder (AUD). However, only about 8% of adults who had AUD in the past year received treatment. Surveys have also shown racial disparities regarding AUD treatments. Thus, it is imperative to identify racial disparities in AUD patients, as it may indicate a specific underlying pathophysiology in an AUD subpopulation. To identify racial disparity in AUD, we enrolled 64 cohorts, including 26 AUD participants and 38 healthy controls, from Northwest Louisiana using community-based enrollment. Then, we used psychometric scales to assess alcohol drinking patterns and measured blood metabolites change using LC-MS/MS. Alcohol-related scales from the questionnaires did not differ between the Caucasian AUD participants and African-American AUD participants. From blood metabolomics analyses, we identified that 6 amino acids were significantly different by AUD status and or race. Interestingly, Caucasian AUD participants had a higher glutamate metabolism mediated by glutamine synthetase (GS). The correlation between blood glutamate/glutamine ratio and GS activity was only significant in the Caucasian AUD group whereas no changes were observed in African-American AUD group or controls. Taken together, our findings from this sample population demonstrate that blood GS is a potential biomarker associated with Caucasian AUD, which is an important step towards the application of a new pharmacological treatment for AUD.

## Introduction

Alcohol use disorder (AUD) contributes significantly to the global burden of disease measured by Disability-Adjusted Life Year (DALY) (139 million disability-adjusted life-years) [1]. Thus, effective clinical intervention is essential. However, this condition is difficult to treat due to the heterogeneity of AUD, likely involving multiple mechanisms. While a critical step in the effective study and treatment of AUD is the identification of disease subgroups, clear parameters for classification are elusive.

Research on disease subgroups has highlighted the importance of discerning racial disparities, of which there are many in AUD. Many studies have noted racial disparities in factors contributing to AUD as well as its treatment. African-Americans may be particularly vulnerable to the negative effects of economic hardship. Following severe economic losses, African-Americans, compared to other races, have a higher likelihood of developing AUD [2]. Another study looked at whether neighborhood disadvantage, defined by low levels of education, employment, and income below the poverty line was likely to increase the number of problem drinkers or persons with AUD. This study suggested that racial differences may play a role in the development of problem drinking, and African-Americans specifically are more likely to develop AUD than Caucasians even when factors like social/economic hardships are controlled for [3]. However, numerous studies have found that the African-American population is more likely to abstain from alcohol completely, and is more likely to consume less alcohol in a single episode than a Caucasian population [4]. However, there is no evidence whether biological or genetic factors contribute to the development of alcohol craving or excessive alcohol drinking. Thus, it is necessary to further investigate biological factors in the context of racial disparity. Moreover, it is important to incorporate a diverse population sample that has African-American AUD individuals.

One innovation of current clinical pharmacology is the use of peripheral metabolites to predict neuropharmacological outcomes against neuropsychiatric disorders [5, 6]. This approach suggests that a genetic variant and/or alcohol drinking pattern possibly modifies amino acid metabolism in both the peripheral and neuronal nervous systems, and possibly in parallel. Importantly, the LC/MS/MS-based metabolomics method is clinically applicable to detect an individual’s metabolic profile; this includes profiles from a healthy or disease condition, and signature metabolites can be used to strengthen both diagnostic and therapeutic predictions [7]. Using this method, the current study demonstrates interesting points regarding glutamate biology in AUD. We hypothesize that increased glutamate regulation may be associated with clinical scales, both of which can help to provide more information about an individual’s clinical diagnosis and on a larger scale, to identify disease subpopulations of AUD [8].

Specifically, the pathophysiological development of AUD has been associated with an imbalance between excitatory and inhibitory neurotransmitters and the subsequent receptor-mediated signaling cascades in the brain [9]. Alterations in the balance between glutamate and GABA levels have been demonstrated to be major contributors to AUD [10]. Among several FDA-approved medications for AUD treatment [11], the anti-glutamatergic medication, acamprosate, increases the time to relapse and helps to maintain abstinence, due to its ability to stabilize glutamatergic imbalances in the brain [12, 13]. However, the treatment outcome is not universal, as it appears that acamprosate may only work in select subpopulations of individuals with AUD [14]. Using pharmacometabolomics, elevated baseline serum glutamate levels mediated by glutamine synthetase (GS) proved to be a potential biomarker associated with positive acamprosate response [8].

In this study, we assessed whether there is any difference in glutamate metabolism between Caucasian and African-American AUD participants. First, we examined psychometric scales including alcohol craving, alcohol intake, drinking pattern, anxiety, and depression to identify possible racial disparities among 64 AUD study participants from community-based recruitment. Using an LC-MS/MS-based metabolomics platform, 16 amino acid metabolites were profiled to determine metabolic differences due to AUD diagnosis and or race. Overall, our findings will provide the framework for developing criteria that can be used to determine AUD subpopulations based on metabolic profiles and clinical scales. Implementing such a method to research psychiatric disease may help to identify disease subpopulations, including those who may have a high probability of benefiting from certain medication treatments based on their individual pathophysiology.

## Materials and methods

### Recruitment of participants for AUD

Sixty-four study participants were recruited through flyer advertisements in the Shreveport and Bossier areas located in the Northwest Louisiana region. Twenty-six participants were identified as meeting the AUD criteria and identified as being Caucasian (*n* = 11) or African-American (*n* = 15). Thirty-eight control participants included Caucasians (*n* = 16) and African-Americans (*n* = 22) who indicated no AUD symptoms. Participants with unstable medical or psychiatric conditions including suicidality, psychosis, severe renal or liver function impairment were excluded from the study. Control subjects included were considered to be normal and healthy individuals with no major psychiatric illnesses or AUD diagnoses. The inclusion criteria for AUD subjects were male or female patients, aged 21–55, with a diagnosis of AUD based on the Diagnostic and Statistical Manual of Mental Disorders-5 (DSM-5) and confirmed with Alcohol Use Disorders Identification Test (AUDIT-C) [15]. Two female Caucasian AUD participants (2/11) and three female African-American AUD participants (3/15) were enrolled. Two Caucasian AUD participants (2/11) and four African-American AUD participants (4/15) were previously diagnosed with AUD and had a history of AUD clinical treatment. Five Caucasian AUD participants (5/11) and four African-American AUD participants (6/15) had alcoholics anonymous (AA) fellowship history (Table 1). Exclusion criteria included inability to provide informed consent, unstable medical or psychiatric conditions, history of stroke, significant head trauma, brain surgery, or diagnosis of active substance abuse other than alcohol or nicotine. AUD participants with moderate to severe impairments in renal or hepatic functions were not included in the study. Participants should not have consumed alcohol within 24 h prior to beginning the study nor present active signs of severe alcohol withdrawal during the study. Subjects taking AUD medications or anti-psychotic medications at the time of the study were excluded. Informed consent was obtained from all the participants. This study was approved by the Institutional Review Board (IRB000892, PI: Nam H.W.) of Louisiana State University Health Sciences Center Shreveport and was conducted according to the Code of Ethics of the World Medical Association (Declaration of Helsinki).

**Table 1:** Demographic statistics of Caucasian AUD participants and African-American AUD participants.

	Sex	Age (Mean ± SD)	Clinical treatment	AA fellowship	Non-treated	Alcohol withdrawal (DSM-5)
Caucasian AUD (*n* = 11)	M: 9 F: 2	46.64 ± 6.51	18.2% (2/11)	45.4% (5/11)	36.3% (4/11)	54.5% (6/11)
African-American AUD (*n* = 15)	M: 12 F: 3	49.33 ± 6.80	26.6% (4/15)	40.0% (6/15)	33.3% (5/15)	86.7% (13/15)

### Study design and outcome measures

All potential AUD subjects were initially screened by phone using the AUDIT-C as a way to quantify their general alcohol intake and gauge their eligibility as an AUD participant. During the scheduled appointment, in-person interviews were conducted by psychiatrists to determine AUD according to the DSM-5 criteria. All study participants were able to provide informed consent with no apparent compromise of judgement due to any alcohol use on the day of study entry and had no active signs of severe alcohol withdrawal. Timeline Follow Back (TLFB-7 days) [16], Penn Alcohol Craving Scale (PACS) [17], Inventory of Drug-Taking Situations (IDTS-8) [18], Patient Health Questionnaire (PHQ-9) [19], and Generalized Anxiety Disorder Assessment (GAD-7) [20] were self-reported and scored by study coordinators. All data pertaining to the study participant was deidentified and labeled with their corresponding study participant identification number.

### Biospecimen collection and methods

Biospecimens from each subject were collected after administering the psychometric tests. Venipuncture was performed using standard techniques. A total of 12 ml of blood was collected from each subject during the course of the study. All tubes were deidentified with their study participant identification number. After collection, samples were placed on ice until being processed in the lab. There, samples were positioned upright at room temperature for 15–20 min and subsequently spun down for 10 min at 1500 × *g* at 4 °C. Serum and plasma were aliquoted into 250 μl samples and stored at −80 °C within 2 h to minimize any possible metabolite degradation. All samples were thawed on ice for −2 h before use.

### Pharmacometabolomics using LC-MS/MS

Serum amino acid calibration standards were prepared with AccQ•Tag™ Ultra Amino Acid Analysis Solution (AA) kit from Waters according to instructions with slight modifications for detection on a mass spectrometer [21]. A 5-point standard concentration curve was made from the calibration standard solution to calculate amino acid concentrations in serum samples. Serum samples of 10 μl were spiked with an internal standard, norvaline then derivatized according to AccQ•Tag instructions. High-resolution separation was done using an ACQUITY UPLC system and injecting 1 μl, with an Amino Acid Analysis column from Waters. Mass detection was completed on a XEVO TQ-S Mass Spectrometry, Waters in ESI positive mode.

### GS enzyme assay

Plasma GS activity was measured by a colorimetric enzyme assay kit (BioVison, Milpitas, CA). Ten microliters of plasma sample from each participant was prepared in a 96-well plate and conditioned with assay buffers. Based on the manufacturer’s instructions, we conducted GS-mediated hydrolysis from Glutamate to Glutamine and ADP. Then, we measured ADP production in the subsequent enzymatic reaction in the presence of ADP Converter, ADP Developer Mix, and ADP Probe, which ultimately forms a colorimetric product that is measured at OD 570 nm. Enzyme activity was calculated as U units based on the standard calibration curve.

### Statistical analysis

Data are described as mean ± SEM. Statistical analyses were performed using Prism (v 8.00 GraphPad Software, La Jolla) and SigmaPlot (v13, SYSTAT Software, Point Richmond). Each data was analyzed using the Shapiro-Wilk test to confirm the normality of data distribution. For parametric analysis, unpaired *t*-test or ANOVA were used. For non-parametric analysis, Mann–Whitney test or Kruskal–Wallis test were used. Logistic regression analysis, receiver operating characteristic (ROC) curve analyses and the areas under curve (AUCs) were analyzed by JMP16 (SAS, Cary, NC). Results were considered significantly different when *p* < 0.05. For the metabolomics profiling, the significance was adjusted for multiple comparisons using Bonferroni correction *p* value < 3.0 × 10^−3^ (0.05/16).

## Results

### Clinical characteristics of Caucasian AUD and African-American AUD participants

For this racial disparity study, a total of 64 participants were enrolled by community-based recruitment. AUD participants were diagnosed with the Diagnostic and Statistical Manual of Mental Disorders-5 (DSM-5) and confirmed with Alcohol Use Disorders Identification Test (AUDIT-C). Twety-six participants were identified as meeting the AUD criteria and identified as being Caucasian (*n* = 11) or African-American (*n* = 15). Demographic information for AUD participants is presented in Table 1. A greater proportion of AUD participants were male. There was no significant age difference between the Caucasian AUD participants and African-American AUD participants. 18.2% of Caucasian AUD participants (2/11) and 26.6% of African-American AUD participants (4/15) had a prior clinical diagnosis of AUD and medication treatment history. 45.5% of Caucasian AUD participants (5/11) and 40.0% of African-American AUD participants (6/15) had a history of AA fellowship. Control participants included Caucasians (*n* = 16) and African-Americans (*n* = 22) who indicated no AUD symptoms or any psychiatric concerns.

**Fig. 1:**
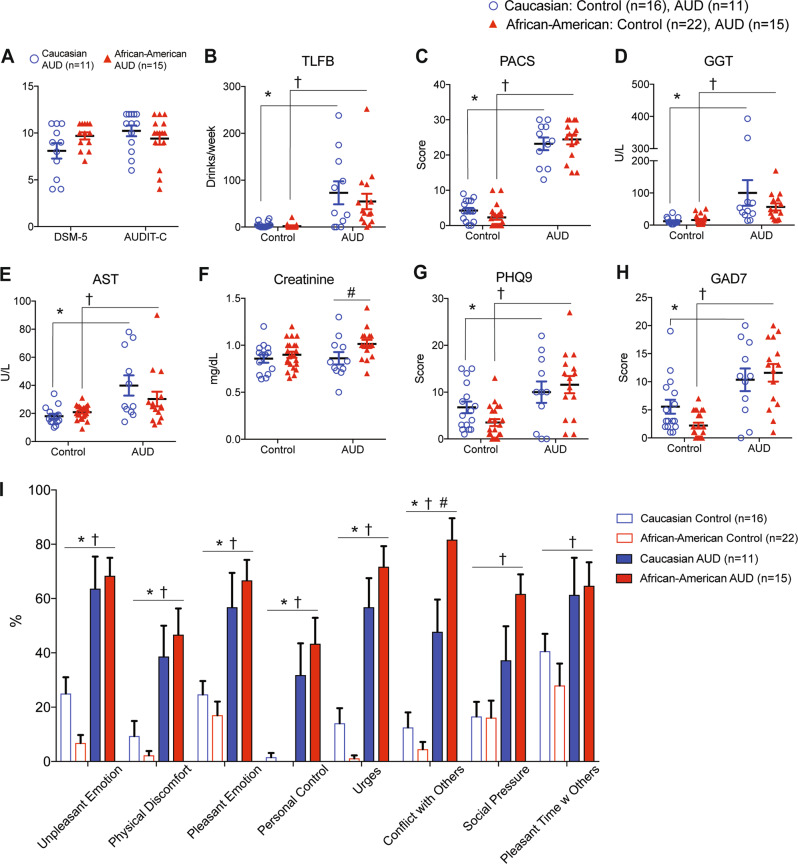
Clinical characteristics of Caucasian AUD and African-American AUD participants. **A** AUD participants were diagnosed with the DSM-5 and AUDIT-C. There were no significant differences between AUD groups in terms of their disease severity. **B** Alcohol consumption measured by Timeline Follow Back-7 days (TLFB-7) is significantly increased in both AUD groups. **C** Alcohol craving measured by Pennsylvania Alcohol Craving Scale (PACS) is increased in both AUD groups. Both TLFB-7 and PACS were significantly different between control and AUD participants but not significant between Caucasian AUD and African-American AUD participants. **D**, **E** Liver damage markers, gamma-glutamyl transferase (GGT) and aspartate aminotransferase (AST), were significantly higher in AUD groups compared to control participants. **F** Blood creatinine levels were in the normal range in both control and AUD participants, but Two-way ANOVA indicates significantly increased creatinine levels in African-American AUD participants compared to that of Caucasian AUD participants (^#^*p* < 0.05). **G**, **H** Symptoms of depression (measured by patient health questionnaire; PHQ9) and anxiety (measured by generalized anxiety disorder; GAD7) were significantly higher in AUD subjects compared to controls. **I** Inventory of Drug-Taking Situations (IDTS-8) provides a profile of eight types of high-risk situations in which a participant has used alcohol over the past year. All data are presented as mean ± SEM. Statistics was by two-way ANOVA followed by the Tukey post hoc test. *p < 0.05 indicates statistical significance between Caucasian AUD and Caucasian control. †*p* < 0.05 indicates statistical significance between African-American AUD and African-American control. ^#^*p* < 0.05 indicates statistical significance between Caucasian AUD and African-American AUD.

Both Caucasian and African-American AUD participants did not differ significantly in their clinical diagnosis by DSM-5 and AUDIT-C (Fig. 1A). From the DSM-5 questionnaire, 86.7% of African-American AUD participants reported having withdrawal syndrome or drinking alcohol to avoid withdrawal in the last year, while 54.5 % of Caucasian AUD participants experienced alcohol withdrawal (Table 1). Two-way ANOVA indicates that alcohol consumption (measured by total drinks in the past 7 days by Time Line Follow Back-7 (TLFB-7)) was significantly different between the control group and AUD group (*F*_(1,60) _= 26.69, *p* < 0.0001), but not significantly different between the Caucasian AUD participants and African-American AUD participants (Fig. 1B). To measure alcohol craving differences in participants, the Pennsylvania Alcohol Craving Scale (PACS) was used [17]. Two-way ANOVA indicates significant differences in PACS scores between control and AUD participants (*F*_(1,60) _= 349.3, *p* < 0.0001), and consistently, no differences in alcohol craving intensity between races in the AUD group (Fig. 1C).

To clinically confirm alcohol use in AUD participants, liver damage and kidney damage markers were examined. AUD participants showed significantly increased gamma-glutamyl transferase (GGT; (*F*_(1,60) _= 17.07, *p* = 0.0001)) and aspartate aminotransferase (AST; (*F*_(1,60) _= 16.46, *p* = 0.0002)) levels compared to those of control participants (Fig. 1D, E). Blood creatinine levels indicating kidney function were in the normal range when comparing between control and AUD participants, but two-way ANOVA identified significantly increased creatinine in African-American AUD participants (*F*_(1,60) _= 4.74, *p* = 0.033) compared to Caucasian AUD participants (Fig. 1F), which is consistent with a previous report [22]. Overall, no racial disparities between Caucasian AUD and African-American AUD participants were detected in alcohol craving, drinking patterns, and liver function related to chronic alcohol use.

### Negative emotion promotes alcohol drinking in African-American AUD participants

Next, we examined whether depression or anxiety contributes to excessive alcohol drinking in Caucasian and African-American AUD participants differently. Intensity of depression (measured using the Patient Health Questionnaire scale, PHQ-9) and anxiety (measured using the Generalized Anxiety Disorder scale, GAD-7) symptoms in AUD participants compared to control participants were measured. Two-way ANOVA identifies that AUD participants showed significantly increased PHQ-9 (*F*_(1,60) _= 15.22, *p* = 0.002) and GAD-7 (*F*_(1,60) _= 31.61, *p* < 0.001) scores compared to control participants (Fig. 1G, H). These findings implicate that chronic alcohol use is increasingly correlated to the intensity of depression and anxiety, of which there are no racial differences. To assess the situational antecedents to the use of alcohol in the past year between Caucasian AUD participants and African-American AUD participants, Inventory of Drug-Taking Situations (IDTS-8) was examined (Fig. 1I). AUD participants indicated significantly increased responses to all 8 subscales compared to control participants. Among 8 subscales, conflict with others (80%) was the most frequent trigger situation that triggered heavy drinking in the African-American AUD group, while unpleasant emotions (62%) was the most frequent situation that triggered alcohol drinking in the Caucasian AUD participants. All situations were significant for African-American AUD participants compared to African-American control participants. All situations except social pressure and pleasant time with others were significant for Caucasian AUD compared to Caucasian control participants. Conflict with others was the only racial difference that was significant between the African-American AUD group and Caucasian AUD group.

### Multivariable analysis of psychometric scale and racial difference

**Fig. 2:**
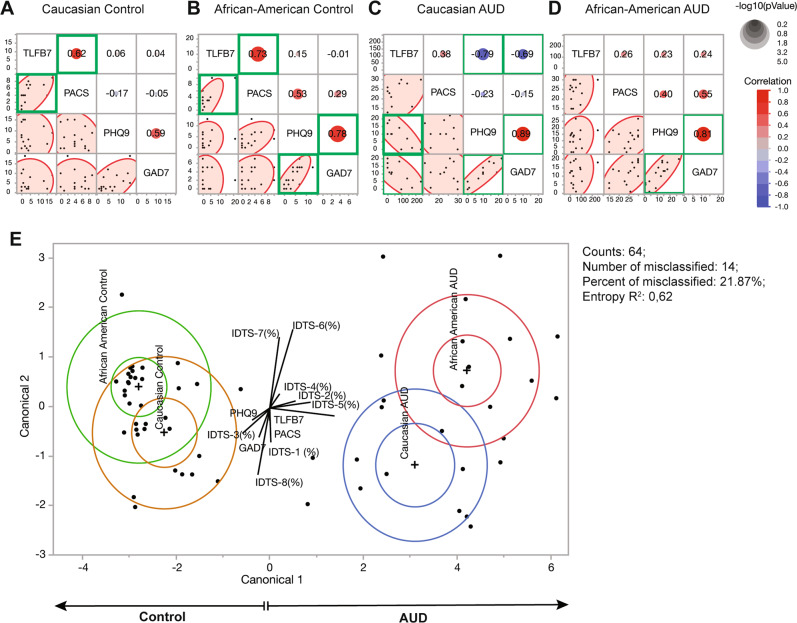
Multivariable analysis of psychometric scales across groups. This regression model depicts the association between alcohol craving, drinking levels, depression, and anxiety across race and AUD diagnosis. **A** Alcohol craving (PACS) and alcohol drinking (TLFB7) showed a significant positive correlation (*R* = 0.62, *p* = 0.004) in Caucasian controls. **B** In African-American control group, alcohol craving (PACS) and alcohol drinking (TLFB7) showed a significant positive correlation (*R* = 0.73, *p* = 0.001). Significant positive correlations were also found between anxiety and depression in this sample group (*R* = 0.78, *p* = 0.001). **C** Significant negative correlation between alcohol intake (TLFB) and depression (*R* = −0.79, *p* = 0.001) as well as anxiety (*R* = −0.69, *p* = 0.001) for the Caucasian AUD group, which is not present in African-American AUD group. **D** African-American AUD groups showed a significant positive correlation (*R* = 0.81, *p* = 0.001) between depression and anxiety. Red circle represents positive correlation, while blue circle represents negative correlation between the *x*-axis and *y*-axis. Circle size indicates *p*-values. Green boxes indicate the statistically significant correlation (*p* < 0.05; correlation coefficient of >0.60). The correlations were estimated by Row-wise method. **E** Principle component analysis (PCA) of psychometric scales can clearly discriminate healthy controls from AUD subjects across the *x*-axis, but not by race. The algorithm was not able to distinguish race accurately, as seen by the overlapping of race clusters and misclassified study subjects indicated by the dots.

Then multivariable logistic regression models were used to evaluate the association between race and psychometric scales of alcohol craving, drinking levels, depression, and anxiety intensity. In the Caucasian control group, alcohol craving (PACS; *y*-axis) and drinking (TLFB-7; *x*-axis) indicated a significant positive correlation (red circle; *R* = 0.62, *p* = 0.004) as expected in healthy social drinkers (Fig. 2A). In the African-American control group, alcohol craving (PACS; *y*-axis) and drinking (TLFB-7; *x*-axis) also indicated significant positive correlation (red circle; *R* = 0.73, *p* = 0.001). In addition, depression (PHQ-9; *x*-axis) and anxiety (GAD-7; *y*-axis) shows a significant positive correlation (red circle; *R* = 0.78, *p* = 0.001) (Fig. 2B). The Caucasian AUD group showed a significant negative correlation (blue circle) between alcohol drinking (TLFB-7; *x*-axis) and depression (PHQ-9; *y*-axis, *R* = −0.79, *p* = 0.001), as well as anxiety scales (GAD-7; *y*-axis, *R* = −0.69, *p* = 0.001). There is also a positive correlation (red circle; *R* = 0.89, *p* = 0.001) between depression (PHQ-9; *x*-axis) and anxiety (GAD-7; *y*-axis) (Fig. 2C). Interestingly, the African-American AUD group only showed a positive correlation (red circle; *R* = 0.81, *p* = 0.001) between depression (PHQ-9; *x*-axis) and anxiety scale (GAD-7; *y*-axis) (Fig. 2D). Overall, our multivariable logistic regression model demonstrated that the Caucasian AUD group shows a relationship between heavy drinking and decreased symptoms of depression and anxiety, which are contrary to observations in both the African-American AUD group and the control groups.

To further investigate racial disparity in AUD, we employed discriminant analysis for alcohol-related psychometric scales against four participant groups. Using the psychometrics data from 64 cohorts, scatterplots for principal component analysis (PCA) were presented, and four clusters based on race and AUD were identified (Fig. 2E). Notably, controls and AUD diagnosis can be discriminated horizontally (canonical 1). However, race cannot be discriminated vertically (canonical 2). Statistical analysis identified that 14 cohorts (21.87%) are misclassified. Overall, the entropy *R*^2^ is 0.62, thus, this method of psychometric scale-based discrimination across groups was not significant.

### Blood metabolites profiling for racial disparity in AUD

**Fig. 3:**
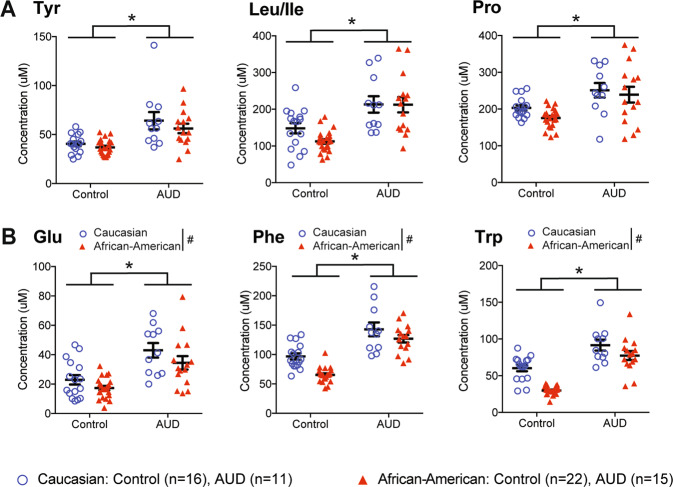
Metabolites profiling for racial disparity in AUD. **A** Tyr, Leu/Ieu, and Pro levels in both races of AUD participants are significantly increased compared to healthy controls. **B** Glu, Phe, and Trp levels are significantly increased in AUD groups compared to controls and Caucasian participants compared to African-American participants. Statistical significance of amino acid levels in response to AUD condition (*p < 0.05) and race (^#^*p* < 0.05) were assessed by two-way ANOVA. Caucasian control (*n* = 16), Caucasian AUD (*n* = 11), African-American control (*n* = 22), and African-American AUD (*n* = 15).

To identify possible blood metabolites that differ between Caucasian and African-American AUD participants, we monitored 16 amino-acid metabolites using UPLC-MS/MS [23]. Significant differences between amino acids were assessed by two-way ANOVA and post hoc tests with Bonferroni correction for multiple comparisons (*p* < 0.003). Two-way ANOVA indicates that six metabolite levels were significantly changed between AUD and control groups. First, AUD participants showed significant differences in metabolites Tyr, Leu/Ile, and Pro compared to those of control participants (Fig. 3A and Supplemental Table 1). AUD participants present 28.2% of the variation in Tyr (Adjusted *R*^2^: coefficient of determination = 0.28), and it is significantly useful in explaining Tyr, *F*_(1, 62)_ = 25.74, *p* < 0.001. Compared to controls, we expect the AUD group to have higher Tyr levels by 21.14 μM, on average (*p < 0.001). AUD explains 32.3% of the variation in Leu/Ile (Adjusted *R*^2^: coefficient of determination = 0.32), and it is significantly useful in explaining Leu/Ile, *F*_(1, 62)_ = 31.08, *p < 0.001. AUD participants indicated higher Leu/Ile levels by 85.20 μM compared to controls. AUD participants presented 21.2% of the variation in Pro (Adjusted *R*^2^: coefficient of determination = 0.21), and showed significant changes in Pro levels, *F*_(1, 62)_ = 17.95, *p < 0.001.

Second, three amino acids showed a statistical significance by AUD diagnosis with a main effect in race (Fig. 3B and Supplemental Table 1). The multiple linear regression model explains 34% of the variation in Glu (Adjusted *R*^2^: coefficient of determination = 0. 34), and it is significantly useful in explaining blood Glu change, *F*_(2, 61)_ = 17.23, *p* < 0.001. Compared to controls, AUD groups had higher Glu by 18.40 μM, on average, keeping race constant (*p < 0.001). Caucasian participants show increased Glu (6.82 μM) compared to African-American participants, on average, at the same level of AUD (^#^*p* = 0.045). Multiple linear regression identifies 61% of the variation in Phe (Adjusted *R*^2^: coefficient of determination = 0. 627), and it is significantly useful in explaining Phe level changes, *F*_(2, 61)_ = 50.31, *p* < 0.001. Compared to controls, AUD show increased Phe (55.01 μM), on average, keeping race constant (*p < 0.001). Compared to Caucasian participants, we would expect African-American participants to show lower Phe (25.10 μM), on average, at the same level of AUD (^#^*p* < 0.001). Multiple linear regression identifies 62.7% of the variation in Trp (Adjusted *R*^2^: coefficient of determination = 0.627), and it is significantly useful in explaining Trp, *F*_(2, 61)_ = 53.84, *p* < 0.001. Compared to controls, we expect AUD participants to have higher Trp by 40.75 μM, on average, keeping race constant (*p < 0.001). Compared to Caucasian participants, we expect African-American participants to have lower Trp by 23.84 μM, on average, at the same level of AUD (^#^*p* < 0.001). These metabolomics results demonstrate a possible racial difference between Caucasian and African-American AUD participants that may elucidate an AUD subpopulation with different pathophysiology.

### GS-dependent glutamate metabolism in Caucasian AUD participants

**Fig. 4:**
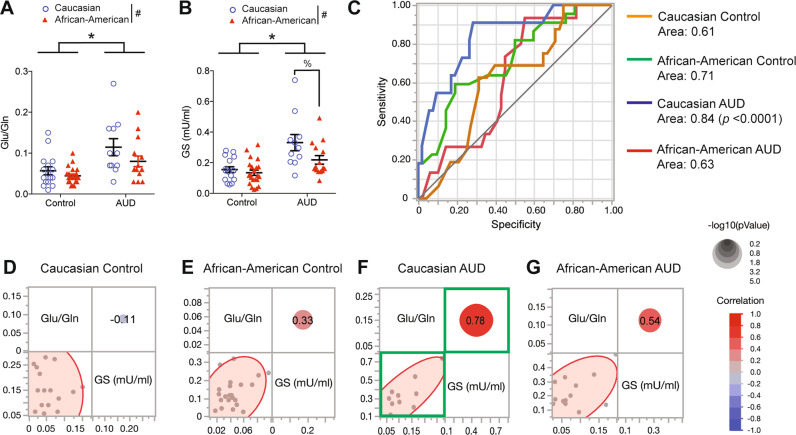
Increased glutamine synthetase-dependent glutamate metabolism in Caucasian AUD participants. **A** Glu/Gln ratio are significantly increased in Caucasian AUD participants. **B** Glutamine synthetase (GS) activity of Caucasian AUD participants is significantly increased compared to other groups. Statistical significance of GS activates in response to AUD condition (*p < 0.05) and race (^#^*p* < 0.05) were assessed by two-way ANOVA. GS activity difference between Caucasian AUD groups and African-American AUD groups was measured by the Mann–Whitney test (%*p* < 0.05). **C** GS levels were the best indicator of the Caucasian AUD group with 0.84 area under the receiver-operating characteristics (ROC) curve. The blue line (Caucasian AUD) on the outer-most part of the graph measures the largest area under the curve and demonstrates high sensitivity and specificity of GS. All data are presented as mean ± SEM. **D**, **E** There is no association between Glu/Gln ratio (*x*-axis) and GS (*y*-axis) in Caucasian control and African-American control group. **F**, **G** There is a significant positive correlation between Glu/Gln ratio and GS activity in Caucasian AUD group (red circle; *R* = 0.78, *p* = 0.001) whereas there is no significant correlation in African-American AUD. The correlations were estimated by Row-wise method. Green boxes indicate the statistically significant correlation and the corresponding graph in which the scores of individual subjects are plotted (*p* < 0.05; correlation coefficient of >0.60).

Of special importance are changes in levels of metabolites involved in glutamate metabolism in AUD. Moreover, increased glutamate/glutamine (Glu/Gln) ratio indicates a perturbation of the glutamate-glutamine system in the brain and has been implicated in alcohol withdrawal [24–26]. Elevated Glu/Gln ratios in the brain are decreased by the anti-glutamatergic medication, acamprosate [27, 28]. Consistently, higher blood glutamate levels prior to treatment are a drug efficacy marker for the anti-glutamatergic medication treating in human AUD [8]. Multiple linear regression identifies 23% of the variation in Glu/Gln (Adjusted *R*^2^: coefficient of determination = 0.23). Two-way ANOVA identifies that blood Glu/Gln is significantly increased in the AUD group (*F*_(1,60) _= 17.17, *p < 0.0001) compared to control groups and in both Caucasian groups compared to both African-American groups (*F*_(1,60) _= 4.35, *p* = 0.04) (Fig. 4A and Supplemental Table 1).

Among different pathways of blood glutamate metabolisms, glutamate transformation to glutamine mediated by GS [29] is an important pathway implicated in the hyper-glutamatergic condition of AUD [30]. Therefore, we measured GS enzyme activity to determine whether this pathway is relevant to glutamate metabolism in our AUD sample population. Multiple linear regression identifies 26% of the variation in GS activity (Adjusted *R*^2^: coefficient of determination = 0.26). Two-way ANOVA identifies that GS activity is significantly increased in the AUD groups (*F*_(1,60) _= 22.11, *p < 0.0001) and Caucasian groups (*F*_(1,60) _= 5.85, ^#^*p* = 0.001). Mann–Whitney test indicates that GS activity is significantly increased in Caucasian AUD groups compared to African-American AUD groups (% *p* = 0.039) (Fig. 4B and Supplemental Table 1). Overall, we found that the serum GS activity was significantly increased in the Caucasian AUD participants compared to their respective race controls and African-American AUD participants. Then, to determine the role of GS activity in AUD and racial disparity, the receiver-operating characteristics (ROC) curve analysis was conducted. Notably, blood GS levels are the best indicator of the Caucasian AUD group with 0.84 area under the curve (Fig. 4C). This result indicates that GS is a powerful discriminator between AUD and race with significant specificity and sensitivity.

Both Glu/Gln ratio and GS activity were significantly increased in the blood of AUD participants, especially Caucasian AUD participants. A multivariable comparison was conducted to determine the association between blood glutamate metabolism and GS in Caucasian AUD. The multivariable comparison identified that there is no significant correlation between Glu/Gln ratio and GS activity in control groups (Fig. 4D, E). Notably, the Caucasian AUD group indicates a significant positive correlation between Glu/Gln ratio and GS activity, while this association is lacking in the African-American AUD group (Fig. 4F, G). These findings based on our sample population suggest that the glutamate metabolism mediated by GS is more dominant in the Caucasian AUD groups.

**Fig. 5:**
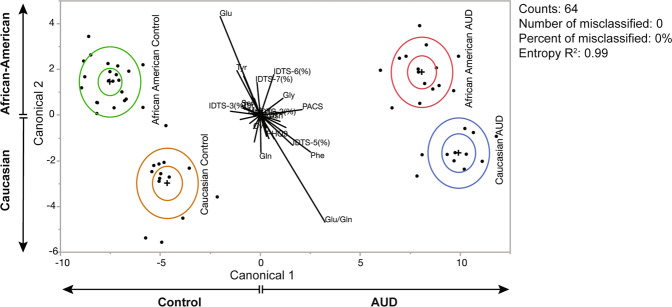
Principle component analysis (PCA) of race differences using a combination of psychometric scales and blood metabolite profiles. PCA shows that data from psychometric scales in conjunction with metabolites can clearly discriminate between clinical diagnosis of AUD (*x*-axis) as well as race (*y*-axis), including between the two categories of specific race and diagnosis. The algorithm was able to distinguish the four participant groups accurately, as there is a lack of overlapped clusters and no misclassified study subjects.

Finally, we employed discriminant analysis using both psychometric scales and 16 blood metabolite levels to discriminate four subject groups. PCA indicated that control and AUD diagnosis can be discriminated horizontally (canonical 1; *x*-axis) and by race, discriminated vertically (canonical 2; *y*-axis). Statistical analysis identified that 100% of the cohorts were classified (i.e., no individuals were misclassified) with the entropy *R*^2^ being 0.99. This result indicates that racial disparity is existent in AUD and can be identified when both psychometric scales and blood metabolites are incorporated (Fig. 5).

## Discussion

Our study demonstrated a possible biological mechanism of racial differences in AUD subjects. Although there were no differences in psychometric scales between the Caucasian AUD and African-American AUD groups, data from blood metabolites demonstrated significant differences between the Caucasian AUD group and African-American AUD group compared to non-AUD controls. Importantly, GS metabolism was significantly increased in the Caucasian AUD group while the African-American AUD group, as well as control groups, lacked this association. Although this study was conducted in a community-based enrollment with small AUD cohorts, however, our blinded analysis provides adequate statistical power to identify biological changes in the blood [31]. Moreover, multivariable analysis enabled us to investigate the relationships between the metabolic profiles of AUD participants and their psychometric scales. Overall, using blood metabolomics along with psychometric scales can be great prospects for ultimately finding biomarkers related to AUD subpopulations.

Findings from this study may contribute greatly to the current understanding of glutamate biology in AUD and provide more evidence to tackle the glutamate theory of AUD. This study attempts to: elucidate how glutamate is strongly related to alcohol use and whether this relationship is consistent between different races affected by AUD; and determine potential reliable biomarkers to identify clinical subpopulations to aid in the development of individualized AUD treatments. Heavy alcohol drinking can cause alterations in glutamate circuits in the brain, resulting in habit formation and compulsion to use the drug. Specifically, synaptic changes in glutamate-modulated N-methyl-D-aspartate (NMDA) receptors and α-amino-3-hydroxy-5-methyl-4-isoxazolepropionic acid (AMPA) receptors in glutamatergic projections are responsible for neurobehavioral adaptations to heavy alcohol use. Upregulation of glutamatergic projections may create circuits in the brain that react to conditioned cues and cause compulsive use of alcohol in AUD individuals [32].

Our measurement of glutamate metabolism in this study was restricted to peripheral function, mainly mediated by the liver. Our results show that blood glutamate levels and the function of the GS pathway for glutamate metabolism were significantly higher in the AUD participants, whereas GS activity was not associated with glutamate levels were observed in the healthy control group. A previous brain magnetic resonance spectroscopy (MRS) study reported that blood glutamate levels measured by LC-MS were not associated with cortical glutamate levels measured by MRS since glutamate transport to the brain is blocked by the blood-brain barrier. However, the glutamine concentration in the cortex is positively correlated with glutamine in plasma [33]. Nonetheless, the intricate glutamate dynamics between plasma and the brain is crucial for considering possible biomarkers. Therefore, we tried to normalize the data since blood glutamine is positively associated with brain glutamine levels and glutamate-glutamine dysregulation in AUD can be indicated by Glu/Gln ratio.

Furthermore, it is required to investigate whether blood Glu/Gln ratio or GS activity is correlated with brain Glu/Gln ratio. Since GS is highly expressed in astrocytes [29] and associated with alcohol withdrawal in the brain [34], our findings may help to provide a possible mechanism alleviating a hyper-glutamatergic state in the brain as found in AUD [13, 35]. Notably, a reduction in the serum Glu/Gln ratio in response to the anti-glutamatergic medication, acamprosate, during alcohol withdrawal is consistent with brain glutamate level changes in response to acamprosate, and is observed in both human and rodent brains measured by MRS [27, 28]. Acamprosate is not a uniform pharmacological treatment for AUD. Acamprosate was shown to have a significant beneficial effect in enhancing abstinence in recently detoxified, AUD individuals from European studies [14, 36]. However, acamprosate was ineffective in reducing drinking in the East Asian sample population [37]. The efficacy of acamprosate for African-American AUD patients have never been reported, although the COMBINE study examined the efficacy of naltrexone for the treatment of AUD among African-American AUD patients [38]. The COMBINE study reported that naltrexone treatment is not beneficial to decrease alcohol drinking in the African Americans AUD subpopulation because pharmacogenetic variants of the OPRM1 gene are infrequent among people of African descent. These results and our study findings provide a further need to investigate other possible variabilities between races such as GS and whether its changes in the blood are reflected in the brain consistently across individuals with AUD.

We expect that genetic variants of glutamate or glutamine signaling may differentially affect GS functioning. Indeed, the glutaminase (*GSL1*) genotype is associated with glutamate-glutamine ratios in its alteration in the cortex measured by in vivo MRS [24]. Furthermore, it is important to follow up on why elevated glutamate levels regulated by GS metabolism is predominant in Caucasian AUD because this emphasizes a specific role of GS metabolism in heavy alcohol use. Particularly, in Caucasian individuals with AUD in this study sample, GS activity is positively correlated with Glu/Gln ratio more so than in their African-American AUD and healthy control counterparts, which indicates that the perturbation in the glutamate-glutamine mechanism may be specific to race and AUD diagnosis [26, 27]. While the correlations between GS and Glu/Gln are higher in AUD, interestingly, multivariable modeling identified a significant positive correlation between GS and Glu/Gln only in the Caucasian AUD population. It must be noted, however, that GS does not solely reflect Glu/Gln because GS activity also requires ammonia and ATP as co-factors. Ammonia can further contribute to the discussion on peripheral metabolites influencing central functions. While genetic variations of enzymes can contribute to the ratio of substrate to products, glutamine to glutamate ratio has also been shown to be modified by other factors like ammonia levels, indicating that such indexes that are used to reflect synaptic levels are influenced by non-synaptic metabolites. The Glu/Gln ratio can also differ based on glutaminase activity [24].

Although we did not ask about the history of ICU admission for severe alcohol withdrawal, we could get some information about alcohol withdrawal based on DSM-5 questionnaire. 54% (6/11) of Caucasian participants experienced withdrawal syndrome or alcohol use to avoid withdrawal, while 87% (13/15) of African American participants experienced alcohol withdrawal. Since human MRS studies demonstrated excessive glutamate levels and increased Glu/Gln ratio in the anterior cingulate cortex (ACC) as a potential biomarker during acute alcohol withdrawal [25], it is required to test whether blood GS activity or Glu/Gln ratio can also indicate alcohol withdrawal.

We could not find any association between blood metabolites and alcohol-related psychometric scales. We expected to observe environmental or genetic factors that contribute to alcohol drinking patterns or alcohol craving levels. As shown in Fig. 1, average alcohol consumption (TLFB-7), craving (PACS), liver damage (GGT and AST), depression (PHQ-9), and anxiety (GAD-7) did not differ between the Caucasian AUD and African-American AUD groups. Several AUD studies reported that patients with AUD may have been attempting to self-medicate their depressive symptoms by consuming alcohol [39]. This is also in line with the findings of a 2018 review report that provided several conclusions regarding patients’ use of alcohol to self-medicate for anxiety and mood disorders: first, a large portion of the population, between 21.9% and 24.1%, with anxiety and mood disorders reports using alcohol for relief of their symptoms, and longitudinal studies of these individuals show that they are significantly more likely to develop AUD than those who do not self-medicate [40]. There is a fine line between using alcohol to self-medicate for these disorders and developing AUD; thus, it is unknown whether such disorders further the development of AUD or if AUD contributes to or exacerbates mood disorders. Notably, we observed that Caucasian AUD participants demonstrate a negative correlation between alcohol drinking and depression/anxiety. Therefore, further details should be required to identify the onset of AUD vs. the onset of anxiety or mood disorders in order to examine whether Caucasian AUD groups use alcohol to self-medicate before or after the onset of anxiety and mood disorders. The lack of a clear relationship between the onsets of these disorders suggests that there are multiple pathways for the development of each and eventual comorbidity. Nevertheless, there is strong evidence that patients who do choose to self-medicate with alcohol for both mood and anxiety disorders have a significantly higher risk of developing AUD as a result, and also to experience persistence and maintenance of the AUD once it has developed [41].

Our AUD participants from Northwest Louisiana were selected based on the Alcohol Use Disorders Identification Test (AUDIT-C) and diagnosed clinically by psychiatrists using the Diagnostic and Statistical Manual of Mental Disorders, 5th Edition (DSM-5). We observed that both the Caucasian AUD and African-American AUD groups had significantly increased PHQ-9 and GAD-7 levels compared to the healthy control groups, making our AUD demographic more “atypical” as opposed to ideal AUD participants who only present AUD symptoms and no symptoms of any mood disorders. However, our participants may present a real-world problem observed in our community of individuals suffering from AUD. In addition, our AUD groups presented a correlated pattern between anxiety and depression [42], which was dramatically different to that of the Caucasian control group (Fig. 2). Consistently, patients who had received a diagnosis of both mood disorder and any anxiety disorder are more likely to have an associated severe addictive behavior governed by a dysregulation in the reward circuit. Particularly relevant to mediating reward, the striatum has been demonstrated to be abnormal as well in these two diagnoses. This effect is mostly seen in patients experiencing anhedonia which can be a feature of both depression and anxiety. This provides further evidence that certain functional connectivity within certain circuits for AUD may be similarly altered by depression and anxiety disorders [43]. AUD is associated with most anxiety disorders among Caucasians and African-Americans [44]. Therefore, an alternative approach to treating AUD that may be more beneficial for this disease subpopulation who have comorbid anxiety or depression are medical treatments that target mood disorder-induced AUD. Both mood and anxiety disorders have been well-reported in literature to increase the odds of having a concurrent AUD [45, 46]. A greater understanding of how these three mental pathologies overlap and contribute to one another is important for the development of treatment plans to address all aspects of a patient’s mental health.

The most significant finding from our study is increased GS activity in this sample Caucasian AUD population. Because GS is highly expressed in the cortex and regulates glutamate metabolism, we need to examine brain Glu/Gln ratios in patients with AUD. Several lines of evidence suggest that the anterior cingulate cortex (ACC) is a key brain region that influences alcohol craving [47]. The ACC is strategically positioned to regulate emotions and behavioral conflict and plays a major role in reward-based decision-making for alcohol drinking. On a neurochemical basis, the vast majority of ACC neurons are glutamatergic and its dysregulation during different stages of addiction has been well-documented [48]. Therefore, it is necessary to replicate and validate our finding on a larger scale by measuring brain metabolites using MRS and to determine whether the GS-mediated glutamate to glutamine mechanism is dominant in Caucasian AUD individuals.

Importantly, increased blood and brain glutamate metabolism is known as a predictive biomarker for the FDA-approved anti-glutamatergic medication, acamprosate, for AUD treatment. Acamprosate responder groups demonstrated elevated baseline serum glutamate mediated by GS [8]. The levels of ACC glutamate were also reduced by acamprosate treatment in AUD patients [49]. Therefore, significantly elevated blood glutamate levels mediated by GS in the Caucasian AUD group may implicate acamprosate treatment to be beneficial and possibly at a higher efficacy compared to that for the African-American AUD group if such a study was conducted. Moreover, our finding may suggest that glutamate dysregulation in AUD is more prevalent in the Caucasian population than in African-Americans with AUD. Although some Caucasian AUD participants show normal glutamate levels and some African-American AUD participants show elevated glutamate levels, the correlation between glutamate levels and GS enzyme activity was discriminant between races. The role of GS in AUD has not been studied thoroughly, although it plays an important role in glutamate-glutamine transformation in the astrocytes and ammonia-related liver toxicity. It is unclear why our Caucasian AUD participants demonstrated a significant correlation between blood Glu/Gln and GS levels, while the control and African-American AUD groups lacked such a strong association.

Using blood metabolomics, we also observed significant changes in metabolites between healthy controls and AUD participants regardless of race. Our results are consistent with the literature described previously showing increased blood glutamate levels in chronic alcohol abuse [8]. Dietary phenylalanine and tyrosine serve as a precursor in catecholamine synthesis, notably dopamine. Therefore, the availability of tyrosine influences the body’s ability to produce dopamine [50]. This is particularly important when considering the dopaminergic hypothesis of addiction. Human and non-human primate studies demonstrated that a diet deficient in phenylalanine/tyrosine decreases alcohol self-administration [51, 52]. Consistently, acute phenylalanine/tyrosine depletion reduces motivation to smoke cigarettes across stages of addiction [53]. Several studies have demonstrated that there is a measurable hyperprolinemia associated with a history of alcohol abuse. The elevation in serum proline has been attributed to both an increase in its production from biochemical precursors and a decrease in proline degradation by proline oxidase in the liver [54, 55]. Animal and human models have both demonstrated that chronic alcohol use leads to an increase in the branched-chain amino acids (BCAA), such as leucine [56, 57]. In rats that consumed alcohol chronically, leucine metabolism was altered as demonstrated by tracer-labeled leucine metabolism studies. These metabolic changes include increased leucine turnover, increased oxidation, and decreased leucine incorporation into the liver and muscle. It is not surprising, therefore, that our participants demonstrated altered BCAA metabolism. However, fewer studies have investigated the inverse relationship of leucine on alcohol, i.e. the effects of leucine on the metabolism of alcohol. One such study has demonstrated that leucine accelerates alcohol clearance from the blood via increased activity of the alcohol dehydrogenase (ADH) enzyme [58]. The combined effect of an alcohol-induced increase in serum leucine levels and an increased rate of alcohol metabolism likely results in a self-perpetuating cycle of increased alcohol use to achieve the same level of psychoactive effects that are commonly seen in AUD. Overall, the metabolomics data from 64 cohorts enabled us to obtain both statistical significance and power in assessing possible AUD subpopulations.

Our findings should be considered in the context of the following limitations. First, the use of community-based enrollment means that the study samples collected in our discovery cohorts do not allow for analyses generalizing race-specific differences in AUD. Thus, we need to replicate our GS findings using larger cohorts that are also of different regions throughout the country and using brain imaging techniques such as MRS to relate peripheral metabolites to central metabolites in the brain. Several brain and blood metabolomics studies focusing on glutamate signaling achieved statistical significance using 10–20 cohorts per group [26, 27]. Our study was a feasibility endeavor to identify possible subpopulations in AUD and results suggested that glutamate metabolism may differ between races. Larger sample size will help us to further distinguish the glutamate pathophysiology in Caucasian AUD and African-American AUD. Moreover, we enrolled the control participants to elucidate the factors that could impact chronic alcohol-induced changes in amino acids between races. However, social and economic conditions can be very different between AUD and control groups and within groups as well, and thus, should be considered as a possible limitation of our study. The second potential limitation is that we have only investigated amino acids and their derivatives. Metabolic abnormalities associated with chronic alcohol use have been well-documented and warrant further exploration to assess racial disparities as a way to understand possible pathophysiological mechanisms of AUD. This metabolomics platform can be applied to studying various other diseases and disorders and may give rise to individualized treatment options that cater to different symptoms and disease characteristics. In conclusion, our study demonstrated that GS-mediated glutamate metabolism is significant in the sample Caucasian AUD population compared to that of the African-American AUD participants, and this may provide important information to how the glutamate theory of AUD is applied in establishing a personalized approach to treating AUD.

## Supplementary information

Supplemental Table

## References

[1] Whiteford HA, Degenhardt L, Rehm J, Baxter AJ, Ferrari AJ, Erskine HE (2013). Global burden of disease attributable to mental and substance use disorders: findings from the Global Burden of Disease Study 2010. Lancet.

[2] Zemore SE, Mulia N, Jones-Webb RJ, Liu H, Schmidt L (2013). The 2008-2009 recession and alcohol outcomes: differential exposure and vulnerability for Black and Latino populations. J Stud Alcohol Drugs.

[3] Karriker-Jaffe KJ, Zemore SE, Mulia N, Jones-Webb R, Bond J, Greenfield TK (2012). Neighborhood disadvantage and adult alcohol outcomes: differential risk by race and gender. J Stud Alcohol Drugs.

[4] Zapolski TC, Pedersen SL, McCarthy DM, Smith GT (2014). Less drinking, yet more problems: understanding African American drinking and related problems. Psychol Bull.

[5] Kaddurah-Daouk R, Weinshilboum RM (2014). Pharmacometabolomics: implications for clinical pharmacology and systems pharmacology. Clin Pharmacol therapeutics.

[6] Quinones MP, Kaddurah-Daouk R (2009). Metabolomics tools for identifying biomarkers for neuropsychiatric diseases. Neurobiol Dis..

[7] Wood PL (2014). Mass spectrometry strategies for clinical metabolomics and lipidomics in psychiatry, neurology, and neuro-oncology. Neuropsychopharmacology.

[8] Nam HW, Karpyak VM, Hinton DJ, Geske JR, Ho AM, Prieto ML (2015). Elevated baseline serum glutamate as a pharmacometabolomic biomarker for acamprosate treatment outcome in alcohol-dependent subjects. Transl Psychiatry.

[9] Koob GF, Volkow ND (2010). Neurocircuitry of addiction. Neuropsychopharmacology..

[10] Tsai G, Coyle JT (1998). The role of glutamatergic neurotransmission in the pathophysiology of alcoholism. Annu Rev Med..

[11] Witkiewitz K, Litten RZ, Leggio L (2019). Advances in the science and treatment of alcohol use disorder. Sci Adv..

[12] Schuckit MA (2009). Alcohol-use disorders. Lancet..

[13] Koob GF, Mason BJ, De Witte P, Littleton J, Siggins GR (2002). Potential neuroprotective effects of acamprosate. Alcohol, Clin Exp Res.

[14] Mann K, Lemenager T, Hoffmann S, Reinhard I, Hermann D, Batra A (2013). Results of a double-blind, placebo-controlled pharmacotherapy trial in alcoholism conducted in Germany and comparison with the US COMBINE study. Addiction Biol.

[15] Bush K, Kivlahan DR, McDonell MB, Fihn SD, Bradley KA (1998). The AUDIT alcohol consumption questions (AUDIT-C): an effective brief screening test for problem drinking. Ambulatory Care Quality Improvement Project (ACQUIP). Alcohol Use Disorders Identification Test. Arch Intern Med..

[16] Breslin FC, Borsoi D, Cunningham JA, Koski-Jannes A (2001). Help-seeking timeline followback for problem drinkers: preliminary comparison with agency records of treatment contacts. J Stud Alcohol..

[17] Flannery BA, Volpicelli JR, Pettinati HM (1999). Psychometric properties of the Penn Alcohol Craving Scale. Alcohol, Clin Exp Res.

[18] Turner NE, Annis HM, Sklar SM (1997). Measurement of antecedents to drug and alcohol use: psychometric properties of the Inventory of Drug-Taking Situations (IDTS). Behav Res Ther..

[19] Lowe B, Unutzer J, Callahan CM, Perkins AJ, Kroenke K (2004). Monitoring depression treatment outcomes with the patient health questionnaire-9. Med Care..

[20] Spitzer RL, Kroenke K, Williams JB, Lowe B (2006). A brief measure for assessing generalized anxiety disorder: the GAD-7. Arch Intern Med..

[21] Armenta JM, Cortes DF, Pisciotta JM, Shuman JL, Blakeslee K, Rasoloson D (2010). Sensitive and rapid method for amino acid quantitation in malaria biological samples using AccQ.Tag ultra performance liquid chromatography-electrospray ionization-MS/MS with multiple reaction monitoring. Anal Chem..

[22] Hsu J, Johansen KL, Hsu CY, Kaysen GA, Chertow GM (2008). Higher serum creatinine concentrations in black patients with chronic kidney disease: beyond nutritional status and body composition. Clin J Am Soc Nephrol..

[23] Lanza IR, Zhang S, Ward LE, Karakelides H, Raftery D, Nair KS (2010). Quantitative metabolomics by H-NMR and LC-MS/MS confirms altered metabolic pathways in diabetes. PLoS ONE.

[24] Ongur D, Haddad S, Prescot AP, Jensen JE, Siburian R, Cohen BM (2011). Relationship between genetic variation in the glutaminase gene GLS1 and brain glutamine/glutamate ratio measured in vivo. Biol Psychiatry.

[25] Hermann D, Weber-Fahr W, Sartorius A, Hoerst M, Frischknecht U, Tunc-Skarka N (2012). Translational magnetic resonance spectroscopy reveals excessive central glutamate levels during alcohol withdrawal in humans and rats. Biol Psychiatry.

[26] Thoma R, Mullins P, Ruhl D, Monnig M, Yeo RA, Caprihan A (2011). Perturbation of the glutamate-glutamine system in alcohol dependence and remission. Neuropsychopharmacology..

[27] Umhau JC, Momenan R, Schwandt ML, Singley E, Lifshitz M, Doty L (2010). Effect of acamprosate on magnetic resonance spectroscopy measures of central glutamate in detoxified alcohol-dependent individuals: a randomized controlled experimental medicine study. Arch Gen Psychiatry.

[28] Hinton DJ, Lee MR, Jacobson TL, Mishra PK, Frye MA, Mrazek DA (2012). Ethanol withdrawal-induced brain metabolites and the pharmacological effects of acamprosate in mice lacking ENT1. Neuropharmacology..

[29] Brusilow SW, Koehler RC, Traystman RJ, Cooper AJ (2010). Astrocyte glutamine synthetase: importance in hyperammonemic syndromes and potential target for therapy. Neurotherapeutics..

[30] Clarke R, Adermark L (2015). Dopaminergic regulation of striatal interneurons in reward and addiction: focus on alcohol. Neural Plast..

[31] Nofziger C, Papaluca M, Terzic A, Waldman S, Paulmichl M (2014). Policies to aid the adoption of personalized medicine. Nat Rev Drug Discov.

[32] Koob GF, Volkow ND (2016). Neurobiology of addiction: a neurocircuitry analysis. Lancet Psychiatry.

[33] Takado Y, Sato N, Kanbe Y, Tomiyasu M, Xin L, Near J, et al. Association between brain and plasma glutamine levels in healthy young subjects investigated by MRS and LC/MS. Nutrients. 2019;11:1649. 10.3390/nu11071649 PMC6682979 31330962

[34] Miguel-Hidalgo JJ (2006). Withdrawal from free-choice ethanol consumption results in increased packing density of glutamine synthetase-immunoreactive astrocytes in the prelimbic cortex of alcohol-preferring rats. Alcohol Alcohol..

[35] Spanagel R, Rosenwasser AM, Schumann G, Sarkar DK (2005). Alcohol consumption and the body’s biological clock. Alcohol Clin Exp Res..

[36] Mann K, Lehert P, Morgan MY (2004). The efficacy of acamprosate in the maintenance of abstinence in alcohol-dependent individuals: results of a meta-analysis. Alcohol, Clin Exp Res.

[37] Namkoong K, Lee BO, Lee PG, Choi MJ, Lee E (2003). Acamprosate in Korean alcohol-dependent patients: a multi-centre, randomized, double-blind, placebo-controlled study. Alcohol Alcohol..

[38] Ray LA, Oslin DW (2009). Naltrexone for the treatment of alcohol dependence among African Americans: results from the COMBINE Study. Drug Alcohol Depend..

[39] Bolton JM, Robinson J, Sareen J (2009). Self-medication of mood disorders with alcohol and drugs in the National Epidemiologic Survey on Alcohol and Related Conditions. J Affect Disord.

[40] Turner S, Mota N, Bolton J, Sareen J (2018). Self-medication with alcohol or drugs for mood and anxiety disorders: a narrative review of the epidemiological literature. Depression Anxiety.

[41] Crum RM, Mojtabai R, Lazareck S, Bolton JM, Robinson J, Sareen J (2013). A prospective assessment of reports of drinking to self-medicate mood symptoms with the incidence and persistence of alcohol dependence. JAMA Psychiatry.

[42] Hirschfeld RM (2001). The comorbidity of major depression and anxiety disorders: recognition and management in primary care. Prim Care Companion J Clin Psychiatry.

[43] Williams LM (2017). Defining biotypes for depression and anxiety based on large-scale circuit dysfunction: a theoretical review of the evidence and future directions for clinical translation. Depress Anxiety.

[44] Smith SM, Stinson FS, Dawson DA, Goldstein R, Huang B, Grant BF (2006). Race/ethnic differences in the prevalence and co-occurrence of substance use disorders and independent mood and anxiety disorders: results from the National Epidemiologic Survey on Alcohol and Related Conditions. Psychol Med..

[45] Grant BF, Stinson FS, Dawson DA, Chou SP, Ruan WJ, Pickering RP (2004). Co-occurrence of 12-month alcohol and drug use disorders and personality disorders in the United States: results from the National Epidemiologic Survey on Alcohol and Related Conditions. Arch Gen Psychiatry.

[46] Grant BF, Stinson FS, Dawson DA, Chou SP, Dufour MC, Compton W (2004). Prevalence and co-occurrence of substance use disorders and independent mood and anxiety disorders: results from the National Epidemiologic Survey on Alcohol and Related Conditions. Arch Gen Psychiatry.

[47] Cheng H, Kellar D, Lake A, Finn P, Rebec GV, Dharmadhikari S (2018). Effects of alcohol cues on MRS glutamate levels in the anterior cingulate. Alcohol Alcohol..

[48] Silveri MM, Cohen-Gilbert J, Crowley DJ, Rosso IM, Jensen JE, Sneider JT (2014). Altered anterior cingulate neurochemistry in emerging adult binge drinkers with a history of alcohol-induced blackouts. Alcohol Clin Exp Res..

[49] Frye MA, Hinton DJ, Karpyak VM, Biernacka JM, Gunderson LJ, Feeder SE (2016). Anterior cingulate glutamate is reduced by acamprosate treatment in patients with alcohol dependence. J Clin Psychopharmacol..

[50] Fernstrom JD, Fernstrom MH (2007). Tyrosine, phenylalanine, and catecholamine synthesis and function in the brain. J Nutr..

[51] Leyton M, Young SN, Blier P, Baker GB, Pihl RO, Benkelfat C (2000). Acute tyrosine depletion and alcohol ingestion in healthy women. Alcohol Clin Exp Res..

[52] Palmour RM, Ervin FR, Baker GB, Young SN (1998). An amino acid mixture deficient in phenylalanine and tyrosine reduces cerebrospinal fluid catecholamine metabolites and alcohol consumption in vervet monkeys. Psychopharmacology.

[53] Venugopalan VV, Casey KF, O’Hara C, O’Loughlin J, Benkelfat C, Fellows LK (2011). Acute phenylalanine/tyrosine depletion reduces motivation to smoke cigarettes across stages of addiction. Neuropsychopharmacology..

[54] Kershenobich D, Garcia-Tsao G, Saldana SA, Rojkind M (1981). Relationship between blood lactic acid and serum proline in alcoholic liver cirrhosis. Gastroenterology..

[55] Mata JM, Kershenobich D, Villarreal E, Rojkind M (1975). Serum free proline and free hydroxyproline in patients with chronic liver disease. Gastroenterology..

[56] Brown JP, Dissanayake VU, Briggs AR, Milic MR, Gee NS (1998). Isolation of the [3H]gabapentin-binding protein/alpha 2 delta Ca2+ channel subunit from porcine brain: development of a radioligand binding assay for alpha 2 delta subunits using [3H]leucine. Anal Biochem..

[57] Bernal CA, Vazquez JA, Adibi SA (1993). Leucine metabolism during chronic ethanol consumption. Metabolism..

[58] Murakami H, Ito M, Furukawa Y, Komai M (2012). Leucine accelerates blood ethanol oxidation by enhancing the activity of ethanol metabolic enzymes in the livers of SHRSP rats. Amino Acids.

