# Supplementary material for: Blood glutamine synthetase signaling in alcohol use disorder and racial disparity
Source: Transl Psychiatry. 2022 Feb 22;12:71. doi: 10.1038/s41398-022-01837-w (PMC8863875; doi:10.1038/s41398-022-01837-w)
Supplement: Supplementary file 1 — Supplemental Table [file 41398_2022_1837_MOESM1_ESM.docx]

**Supplemental Table 1.**

| Amino Acid Metabolites (μM) | Caucasian Control  (n=16) | Caucasian AUD  (n=11) | *p* value | African-American Control (n=22) | African-American AUD (n=15) | *p* value |
| --- | --- | --- | --- | --- | --- | --- |
| Tyr | 40.6 ± 2.37 | 64.2 ± 8.81 | 0.0056 | 36.9 ± 1.68 | 56.2 ± 4.84 | 0.0001 *** |
| Leu/Ile | 148.2 ± 13.90 | 213.1 ± 22.30 | 0.0152 | 112.4 ± 6.26 | 212.3 ± 20.99 | < 0.0001 **** |
| Pro | 203.3 ± 6.98 | 251.1 ± 19.78 | 0.0151 | 175.4 ± 5.70 | 239.2 ± 21.36 | 0.0018 ** |
| Glu | 22.9 ± 3.18 | 43.00 ± 4.98 | 0.0015 ** | 17.3 ± 1.49 | 34.5 ± 4.55 | 0.0002 *** |
| Phe | 96.7 ± 5.17 | 142.9 ± 11.79 | 0.0005 *** | 65.4 ± 2.71 | 126.9 ± 6.31 | < 0.0001 **** |
| Trp | 60.3 ± 4.11 | 91.6 ± 7.46 | 0.0005 *** | 29.8 ± 1.20 | 77.4 ± 6.07 | < 0.0001 **** |
|  |  |  |  |  |  |  |
| GS Activity | Caucasian Control  (n=16) | Caucasian AUD  (n=11) | *p* value | African-American Control (n=22) | African-American AUD (n=15) | *p* value |
| Glu/Gln ratio | 0.057 ± 0.0096 | 0.115 ± 0.0208 | 0.0103 * | 0.0445 ± 0.0043 | 0.080 ± 0.0129 | 0.0050 ** |
| GS (mU/ml) | 0.155 ± 0.0188 | 0.333 ± 0.0525 | 0.0012 ** | 0.134 ± 0.0173 | 0.217 ± 0.0280 | 0.0115 * |

For the metabolomics profiling, the significance was adjusted for multiple comparisons using Bonferroni correction p value < 3.0 x 10-3 (0.05/16). All data are presented as mean ± SEM. Statistics by unpaired t-test or Mann-Whitney U test between control group and AUD group in each race.
